# Cranial Autonomic Symptoms and Neck Pain in Differential Diagnosis of Migraine

**DOI:** 10.3390/diagnostics13040590

**Published:** 2023-02-05

**Authors:** Beatriz Nunes Vicente, Renato Oliveira, Isabel Pavão Martins, Raquel Gil-Gouveia

**Affiliations:** 1 Neurology Department, Headache Outaptient Clinic, Centro Hospitalar Universitário Lisboa Norte, Hospital de Santa Maria, 1649-028 Lisbon, Portugal; 2 Hospital da Luz Headache Center, Neurology Department, Hospital da Luz, 1500-650 Lisbon, Portugal; 3 Centro de Estudos Egas Moniz, Universidade de Lisboa, 1649-028 Lisbon, Portugal; 4 Center for Interdisciplinary Research in Health, Universidade Católica Portuguesa, 1649-023 Lisbon, Portugal

**Keywords:** migraine, cranial autonomic symptoms, cluster headache, neck pain

## Abstract

Cranial autonomic symptoms and neck pain have been reported to be highly prevalent in migraine, although they are rarely considered in clinical evaluation. The aim of this review is to focus on the prevalence, pathophysiology, and clinical characteristics of these two symptoms, and their importance in the differential diagnosis between migraines and other headaches. The most common cranial autonomic symptoms are aural fullness, lacrimation, facial/forehead sweating, and conjunctival injection. Migraineurs experiencing cranial autonomic symptoms are more likely to have more severe, frequent, and longer attacks, as well as higher rates of photophobia, phonophobia, osmophobia, and allodynia. Cranial autonomic symptoms occur due to the activation of the trigeminal autonomic reflex, and the differential diagnosis with cluster headaches can be challenging. Neck pain can be part of the migraine prodromal symptoms or act as a trigger for a migraine attack. The prevalence of neck pain correlates with headache frequency and is associated with treatment resistance and greater disability. The convergence between upper cervical and trigeminal nociception via the trigeminal nucleus caudalis is the likely mechanism for neck pain in migraine. The recognition of cranial autonomic symptoms and neck pain as potential migraine features is important because they often contribute to the misdiagnosis of cervicogenic problems, tension-type headache, cluster headache, and rhinosinusitis in migraine patients, delaying appropriate attack and disease management.

## 1. Introduction

Migraine is a highly disabling neurological disorder. Although headache is its most frequent symptom, migraine is much more than a simple pain disorder as it involves a cluster of painful and painless symptoms that can occur before, during, and after headache [1]. The third edition of the International Classification of Headache Disorders (ICHD-3) characterizes migraine as a unilateral, throbbing, moderate to intense headache, worsened by physical activity, and associated with nausea and/or vomiting, photophobia, and phonophobia [2].

From clinical practice, we know that migraine patients and migraine attacks do not always present these classical criteria-defining migraine features, and several headache experts have been debating whether other nonpainful symptomatology should be included in the classification, looking for possible migraine endophenotypes [3]. The recognition of the whole spectrum of migraine symptoms, beyond headache and aura, has been increasingly recognized, and it is now evident that symptoms can begin hours to days before pain onset and can persist during pain and following pain resolution [1].

Nonpainful symptomatology can be divided into categories, such as sensorial symptoms (photophobia, phonophobia, osmophobia, and allodynia); cranial autonomic symptoms (CAS); mood, sleep, and cognitive changes (drowsiness, irritability, exhaustion, poor memory, and concentration difficulty); homeostatic imbalance (frequent urination, change in bowel habits, and increased thirst and cravings); as well as other migraine-related symptoms, such as neck discomfort or pain, all of which have been reported to be highly prevalent in migraine patients [4]. Increased attention is required to identify such symptoms, in particular neck pain and CAS, as those often contribute to misdiagnosis of cervicogenic problems and rhinosinusitis in migraine patients, often delaying appropriate attack and disease management.

This review will focus on the prevalence, pathophysiology, and clinical characteristics of CAS and neck pain in migraine, and their importance in differential diagnosis between migraine and other headaches.

## 2. Cranial Autonomic Symptoms

### 2.1. CAS as a Migraine Symptom

Extra-cranial autonomic nervous system symptoms, such as cardiovascular autonomic changes, syncopal episodes, temperature dysregulation, urinary symptoms, and gastrointestinal dysfunction, are relatively well described in migraine patients [5].

Nevertheless, CAS-conjunctival injection, lacrimation, nasal congestion, rhinorrhea, miosis, ptosis, facial sweating, facial flushing, eyelid edema, aural fullness, all of which are typically observed in cluster headache (CH) and other trigeminal autonomic cephalalgias (TAC), are rarely considered in the evaluation of migraine [6]. According to several studies, migraine patients with such symptoms are frequently misdiagnosed as sinus headaches and undergo sinus surgeries, which delays an appropriate treatment [7,8].

Migraine with CAS was first reported by Blau in 1970, where vessels from conjunctival, lip, tongue, and nailfold were examined microscopically in 35 migrainous subjects [9].

Although CAS are rarely part of the main complaints of migraine sufferers, the literature shows a wide range of prevalence from 3.1% to 82%, and this rate can be even higher in patients with chronic migraine. This variation is probably explained by different study methodologies and study populations, and most studies have lacked validation of the symptoms [10].

Ocular CAS are more common compared to nasal CAS, probably due to the predominant activation of ophthalmic division of the trigeminal nerve in migraine [7]. The most common CAS reported in different studies are aural fullness, lacrimation, conjunctival injection and facial/forehead sweating [6,7,10], as reported in Table 1. 

Patients with migraine have a lower prevalence of all CAS items compared with CH patients [11]. Recently, an interview-based study with a large sample of migraine patients was made and the prevalence of one and two CAS was 44% and 22%, respectively, and the most common were facial/forehead sweating and lacrimation. The authors of this study proposed a set of diagnostic criteria for migraine with CAS: attacks fulfilling the diagnostic criteria for migraine with or without aura, and 2 reversible CAS must be present (conjunctival injection, lacrimation, nasal congestion, rhinorrhea, forehead or facial sweating, miosis, ptosis, eyelid edema) during at least one third of the attacks [6].

Migraineurs experiencing CAS are more likely to experience more severe, frequent, and longer attacks, especially those who have more than one CAS [10]. However, the presence of CAS does not differ based on age, sex, or presence of aura [5]. In several studies, migraineurs with CAS reported bilateral autonomic symptoms, particularly those with bilateral headaches [7,10,12], and this can be explained by the fact that the superior salivatory nucleus has bilateral innervations from the trigeminal system. Some authors observed that unilaterality of the headache can be a risk factor for the presence of CAS. When evaluating the most severe cases of migraine, CAS tend to lateralize to the side of the headache, similar to what happens in the TAC’s [13]. In addition, higher rates of photophobia, phonophobia, and osmophobia, as well as allodynia have been reported in migraineurs with CAS compared to those without autonomic features [11,12], suggesting that the magnitude of activation of the trigeminal system may imply that it is easier to reach the threshold for triggering the trigeminal autonomic reflex and amplifying the pain [13]. A similar relation with attack severity has been described in cluster headache [18].

The correlation between chronicity of migraine and high prevalence of CAS is not clear from the current literature. There is evidence that CAS appear in around 80% of chronic migraine (CM) patients during attacks and the most frequent symptoms were lacrimation and conjunctival injection, such as in episodic migraine, which were reported by almost half of the patients [19]. However, several other studies examined CAS prevalence separately in patients with episodic migraine and chronic migraine and found no significant difference [11,14,20]. Although it has not yet been validated, a new scale has been designed to evaluate and quantify 5 parasympathetic CAS (lacrimation, conjunctival injection, nasal congestion, eyelid edema, and aural fullness) in primary headaches, and in the future it can be used as a tool when evaluating the response to therapy in clinical trials [19].

This correlation between CAS and migraine is not limited to adults. It is often described in childhood, where as many as 70% of pediatric migraineurs had at least one CAS and most of them had more than one CAS that tended to be bilateral, just as in adults [8]. The most common symptoms in childhood are aural fullness, facial flushing, conjunctival injection and lacrimation, and they seem to be more common in subjects with a higher frequency of migraine attacks, as seen in adults studies [8,21,22]. Recognizing that CAS are common in pediatric migraine is important since sinusitis, which also may present with headache, is one of the most common misdiagnoses given to migraineurs during childhood, putting these patients at an even higher risk of inappropriate treatments for their migraine [8].

Research on therapies for migraine accompanied by CAS is scarce, however, triptans, lidocaine, and onabotulinum toxin A have shown stronger benefits in this group of migraineurs [10]. There is evidence that in individuals with CAS, monoclonal antibodies targeting the calcitonin gene-related peptide (CGRP) pathway greatly decreased migraine headache days, possibly due to increased activation of the trigeminovascular system, reaching a better response to treatment [23].

### 2.2. Pathophysiology of CAS in Migraine

For many years, the “autonomic theory” of migraine pathogenesis has piqued curiosity. However, research has produced contradicting findings. It has been proposed that both sympathetic hypofunction and hyperfunction, as well as parasympathetic hypofunction and hyperfunction, can occur [24]. This lack of consistency is probably due to the differences across the ictal and interictal periods, and the fact that most of these studies have evaluated patients in the interictal period [25]. Most investigations point to a tendency towards sympathetic hypoactivity between episodes and an accentuated sympathetic response during an episode. This can be attributed to repeated ictal stimulation of the sympathetic autonomic system, which leads to downregulation and reduced plasma norepinephrine release, which eventually causes an increase in postsynaptic adrenergic receptors, resulting in ictal hypersensitivity [26].

CAS occur due to the activation of the trigeminal autonomic reflex, a physiological response following harmful stimuli. Pain signals from the trigeminal nerve activate the dorsal raphe nuclei and periaqueductal gray in the brainstem. They are then transmitted to the locus coeruleus in the ipsilateral pons—resulting in activation of the sympathetic system—and then to ipsilateral superior salivatory nucleus, which is the efferent arc responsible for CAS through the activation of the sphenopalatine ganglion and greater superficial petrosal nerve [27]. Postganglionic parasympathetic fibers then leave the sphenopalatine ganglion and act on the lacrimal glands, nasal, and palatal mucosa, resulting in lacrimation, nasal congestion, or rhinorrhea. Activation of this reflex produces a release of vasoactive neuropeptides, mainly CGRP, vasoactive intestinal peptide (VIP), and pituitary adenylate cyclase activating polypeptide, causing vasodilation and perpetuating the pain. Riesco et al. showed that serum levels of VIP correlate with the presence of CAS in migraine, and seem to reflect the activation of the trigeminovascular system [19]. In addition, there is evidence that migraine patients with CAS respond better to triptans, and VIP serum levels were reduced after triptans administration. It can be speculated that the intense activation of the trigeminal autonomic reflex in migraine patients with CAS, led to a stronger recruitment of 5-HT1B/1D receptors, which are the target of triptans [27].

### 2.3. CAS in Other Headaches and Differential Diagnosis

CH together with other TACs, as well as headaches attributed to a disorder of the nose or paranasal sinuses, are two types of headaches that can be associated with CAS. As we mentioned above, CAS are quite common in migraine, so it is relevant to discuss the differential diagnosis between migraine and these two groups.

Activation of the trigeminal vascular system and trigeminal autonomic reflex, as demonstrated by an increase in CGRP and VIP during attacks, are well-established mechanisms involved in pathophysiology of CH and migraine [19]. According to several functional imaging studies, the hypothalamus is involved in the onset of symptoms of these two conditions: the inferior posterior hypothalamic grey matter, ipsilateral to pain, is a critical location in the start of CH, while in migraine, hypothalamic activation occurs both in the prodromal and the ictal phase coupled with the dorsal rostral pons (the so-called brainstem generator) [28].

Both diseases have a genetic background: the risk of first-degree relatives of patients with CH and migraine to develop the above mentioned diseases is, 5–15 times and 3 times higher, respectively, compared to the general population [29]. History of migraine is not uncommon in patients with CH, and both headache disorders can coexist in the same individual. Comorbid migraine is associated with a shorter duration of CH and shorter delay in diagnosing CH. Song et al. reported that 15% of their cohort had comorbid migraine, and the frequency of chronic CH was higher in this group [30].

The key characteristic that sets CH apart from migraine is the temporal pattern: CH attacks last for 15–180 min and occur from one to eight times a day, in series lasting for weeks or months. Although it occurs very rarely (14%), one aspect that can enhance the risk of misdiagnosing CH as migraine is the possibility of the pain switching sides between attacks or cluster periods [31]. Periodicity and circadian rhythmicity can exist in both conditions but are clearly more relevant and frequent in CH. There is evidence that CH and migraine attacks are linked to chronobiological characteristics. In migraine, time preference headache attack (TPHA) may be significantly associated with chronotype: early attacks occur mostly in individuals with early chronotypes, whereas later attacks occurred in individuals with a late chronotype [32]. In CH, some factors are associated with earlier nocturnal peaks in chronorisk: episodic cluster, morning chronotype, good sleepers, not taking verapamil, not smoking, consuming alcohol during the bout, and drinking coffee [33]. These results suggest that to treat migraine and CH patients more effectively, practitioners should be worried about a patient’s chronotype.

One study assessed and characterized CAS in migraine patients versus CH, evincing that 56% of migraine patients experienced CAS, while in CH the percentage was 97% [11]. However, CAS in migraine tend to be less severe and are usually bilateral in contrast to CH patients who, with the exception of facial sweating, exhibit bilateral symptoms only in a small percentage of cases [10]. In CH, CAS can occur either prior or after pain, while in migraine, CAS tend to occur at the peak of pain intensity [6]. While no distinct seasonal pattern has yet been established, one study reported that patients who reported seasonal variation in migraine also reported more CAS and had more severe functional impairment [30].

Along with autonomic symptoms, 25–46% of CH patients also report migraine-related symptoms, such as nausea, photophobia, and phonophobia [30], and there is evidence that, when present, photophobia and phonophobia in TACs appear to be of milder intensity and more lateralized than in migraine [34].

For differentiating chronic migraine from hemicrania continua (HC), the same general principles of laterality and time are considered. The response to indomethacin in HC is an absolute and useful differentiating feature as well. HC patients are nearly three times more likely to have unilateral photophobia or phonophobia, or both, than chronic migraine patients with unilateral pain [34].

Both adult and child migraineurs are frequently misdiagnosed as having “sinus headaches”. In one adult study, 35% of the migraineurs had undergone previous sinus surgery [35]. This was demonstrated by an observational study that included 3000 individuals with a history of self- or clinician-diagnosed “sinus headaches”, in which 88% of these patients met the diagnostic criteria for migraine. However, most of these patients reported additional typical migraine symptoms, such as photophobia, phonophobia, and pulsating head pain [29]. Migraine can be mistaken for sinus headache because of the similarity in location of the headache and the commonly accompanying nasal autonomic symptoms. Pain due to pathology of the nasal mucosa or related structures is usually perceived as frontal or facial, but may be referred more posteriorly, making a proper diagnosis difficult. The presence of purulent nasal discharge and the character of the pain, which is typically pressure-like and reproduced by palpation over the sinuses, help to differentiate these two conditions [5].

## 3. Neck Pain and Migraine

### 3.1. Introduction

While migraine is the second leading cause of disability worldwide, according to the *Global Burden of the Disease 2019* study, neck pain (NP) is also highly prevalent and responsible for 2–6% of the total global years lived with disability [36]. The point prevalence of NP is estimated as 8% and its lifetime prevalence is 48% [37]. Thus, it is not surprising that NP and migraine may present in the same individual just by chance alone. However, in a large American study that included 189,967 adults, NP was self-reported by 38% of individuals with severe headache or migraine, a prevalence much higher than the 11% in individuals without severe headache or migraine [38].

The definition of NP varies between authors and international task forces [39]. The most common definition of NP is a pain located in the anatomical region of the neck with or without radiation to the head, trunk, and upper limbs [40]. NP can arise from many local structures, including muscles, ligaments, facet joints, and visceral structures of the neck.

Initial reports of NP in migraine patients date to more than 3 decades ago, with NP being reported by up to two-thirds of the patients during migraine attacks in the 1980s and 1990s [41,42]. Since then, studies explored the link between migraine and NP from every angle, both from a clinical and research perspective. Yet, it is still an on-going debate whether NP contributes to migraine pain as a trigger, or whether it is a consequence, a part of the migraine attack, or simply a symptom of a co-existing neck disorder.

The recognition of NP as a potential migraine feature is of obvious importance. For example, both adult and pediatric population studies show that misdiagnosis is common, with NP frequently attributed to cervical spine pathology, leading to unnecessary investigations and delay of correct diagnoses [22,43]. Misdiagnosis is more common in migraine patients with pain onset in the cervical region despite these patients still presenting with typical migrainous features [43].

Research on NP and migraine has several concerns and limitations that are worth noticing. First, the assessment of NP differs substantially between studies. In most studies, the prevalence of NP relies on self-administered questionnaires that are subject to recall bias and interpretation difficulties that may result in misclassification, such as localization of pain. In other studies, NP is assessed based on the clinical observation (trigger points, cervical tension), which also has several issues considering the low accuracy of most cervical examination tests [44]. Second, epidemiological studies exploring NP in migraine patients usually exclude confounders, such as cervicogenic headache (CeH) and tension-type headache (TTH). However, as will be discussed later, the overlap between these disorders is significant, particularly if we focus on NP. So, there is potential misdiagnosis bias in the studies. Third, over time, the definitions of concepts, such as premonitory symptoms, have changed as well as diagnostic criteria guidelines, such as ICHD-3 [2,45]. Thus, results may not be directly comparable. Despite appearing to be very common in migraine, NP is an under investigated topic of research [46].

### 3.2. Anatomic and Physiological Considerations and Cervical Musculoskeletal Findings in Migraine

Activation of the trigeminal nerve and its connections is a well-established component of migraine pathophysiology, as discussed above [47]. In addition to the trigeminal nerve, both occipital and cervical afferents converge in the trigeminocervical complex (TCC), which is composed of the C1, C2, and C3 dorsal horns of the cervical spinal cord and the trigeminal nucleus caudalis [48]. Activation of this complex is the mechanism for cervical disease headache as well as for the presence of NP in primary headache disorders, depending on whether it originates in cervical or trigeminal areas [49]. Indeed, there is evidence from experimental studies in healthy individuals that neck muscle induced-pain causes pain in both cervical and trigeminal territories [50].

It is possible that repeated migraine attacks lead to central cervical sensitization and lower pressure pain thresholds associated with increased tension of the neck muscles. The neck muscle stiffness could alternatively be caused directly by alterations in muscles, such as inflammation or trigger points, which may activate sensory neurons and thereby contribute to the development of migraine pain. Another potential mechanism is a dysfunctional descending inhibition in migraine as shown in transcranial magnetic stimulation studies [51]. Further mechanisms include increased stress that may also contribute to prolonged increase of regional muscle tone via the limbic system and the alteration of pain facilitation mechanisms [52]. The well-known interaction between stress and migraine further perpetuates this pain cycle.

Several studies depict cervical musculoskeletal abnormalities and postural changes in different headaches, specifically in migraine [44,46,53]. One of the most frequent findings in migraine patients is the excessive forward head posture (FHP), which means a reduction of the cranio-cervical angle [44]. FHP is thought to result from repeated tasks in the setting of sub-optimal ergonomics and is usually associated with the shortening of the cervical extensor muscles (sub-occipital paraspinals, scalenes, sternocleidomastoid, levator scapulae, upper trapezius, and pectoralis major and minor) [40]. Indeed, a few MRI studies show hyperintense signals in the trapezius in migraine patients [54]. There is also report of the contraction of the scalp muscles, especially temporalis, during migraine attacks due to patients holding or pressing on the affected site for relief [41]. Another common finding in migraine patients is the increased neck muscle stiffness measured by elastography studies [46].

There is also evidence that the pressure pain thresholds in the neck are reduced in migraine patients even when they are not experiencing an attack [46]. Furthermore, one meta-analysis found that pressure pain thresholds measured by quantitative sensory testing (QST) are lower in patients with migraines for the head and neck region but not for the rest of the body [49].

Despite all these data, evidence for cervical musculoskeletal dysfunction in migraine is debatable. Some authors point out that mild impairment in cervical function may be due to variations of the normal, and that differences between healthy controls and migraine patients are only minor [53,55]. It is not clear whether migraine has an associated overall pattern of cervical musculoskeletal dysfunction nor if those changes have clinical meaning and might respond to different therapeutic strategies.

### 3.3. Neck Pain as a Migraine Feature

As stated previously, NP is not part of the ICHD-3 criteria for migraine [45]. However, NP not only seems to be one of the most reported symptoms in migraine, but in some studies NP is more prevalent than classical migraine symptoms, such as nausea [56]. One recent meta-analysis assessing the prevalence of NP in patients with migraine found a pooled relative frequency of 77% in migraine vs. 23.2% in a control group [57]. However, as highlighted by the authors, there is significant heterogeneity among the studies that we must consider in the interpretation of the results.

In most cases, NP is reported before or during a migraine attack (ictal) [37,44,53,56,58]. However, it has been reported after the headache stops (postdromal) and also interictally [41]. In clinical practice, it is often difficult to distinguish between true premonitory and ictal symptoms. Perhaps a more relevant fact is that patients often have difficulties in recognizing premonitory symptoms and mistake them as triggers [59]. Among a cohort of 50 patients with migraine experiencing neck pain, most (89.1%) reported NP only during the migraine pain phase, while 10.9% had NP-triggered migraine attacks [60].

Pharmacological studies with nitroglycerin showed that almost all patients with migraine had at least one premonitory symptom, while 83% went on to experience a full migraine attack [61]. NP was one of those premonitory symptoms showing good agreement for spontaneous and triggered attacks [61]. Furthermore, in an adult population involving 1010 migraine patients, premonitory symptoms (onset of 2 or more hours prior to the headache) were present in 38.9%, the most frequent being a neck tension, phonophobia, and difficulty concentrating [59], which relates very closely to spontaneous complaints in clinical practice.

Migraine sufferers may report NP as the initial site of pain that later radiates forward and reaches criteria for migraine; as a concomitant site of pain during a migraine attack; or as a site of pain following the acute migraine episode. Up to 40% of the migraine patients report that pain starts and/or concentrates in the occipital and neck areas [62]. Regarding gender differences, there are only a few reports showing that women with migraine more frequently report NP during a migraine episode [63].

The presence of NP in migraine has been associated with several clinical and prognostic measures. The prevalence of NP correlates with headache frequency [56]; NP is associated with delayed acute treatment of migraine up to 30 min [64]; NP is associated with treatment resistance and greater disability [56,65]; and NP is more prevalent in chronic migraine than episodic migraine, up to twice the prevalence [57].

Further evidence for NP being part of the migraine cycle comes from studies assessing cervical musculoskeletal function in migraine suffers with NP. First, more than half of those patients have normal neck function [60]. Second, among the patients with cervical dysfunction, cervical dysfunction itself was not associated with pain hypersensitivity, in contrast to NP that was associated with allodynia [60]. Also, neck disability evaluated by The Neck Disability Index seems to be more related to migraine factors including allodynia than to local neck dysfunction [66]. Additional evidence is the interictal reproduction of the typical headache in migraine patients by cervical manipulation [67].

### 3.4. Neck Pain as a Migraine Trigger

Prospective clinical epidemiological studies have reported NP as a trigger in up to 38.4% of migraine patients [68]. In these studies, NP was considered when it was specifically not associated with the migraine pain. Furthermore, NP as a trigger was more common in chronic migraine [68]. In one study, the pressure pain thresholds were similar in migraine patients with and without ictal NP, suggesting a peripheral cause for neck stiffness [46].

Regarding the endurance of the neck muscles, one prospective study showed that migraine patients were more likely to report NP and migraine attacks up to 24 h after the assessment [69]. The authors discuss that the neck soreness and increased nociceptive input from cervical routes triggers the migraine attack. One interesting finding was that not all patients with migraine developed an acute attack after cervical tests, which points out once again to different pain thresholds [69].

Part of the evidence for the neck as a migraine trigger comes from clinical experience, which suggests that reducing neck muscle tension through exercise, physiotherapy, or acupuncture may reduce the frequency of migraine attacks [70]. While the data is scarce, there is evidence of the benefit of massage therapy and physiotherapy on migraine [70]. Addressing postural control impairments is of noted importance as well as the manual treatment of trigger points [70]. Physiotherapy is not necessarily uniform as a preventive drug and should be individualized.

### 3.5. Neck Pain in Other Primary and Secondary Headaches and Differential Diagnosis

The differential diagnosis of NP is extensive and can be challenging. Tension-type headache (TTH) and cervicogenic headache (CeH) are the two main types of headaches associated with the cervical spine and NP. Yet, as we discussed NP is common in migraine, so it is relevant to discuss the differential diagnosis of these disorders.

TTH is usually easy to recognize as a different headache from migraine even when NP is present. In TTH, the pain is not particularly intense and, although relatively common, nausea and photo or phonophobia are less marked than in migraine [45,55]. There are important differences regarding the findings of musculoskeletal dysfunction in these patients. One meta-analysis studied specific cervical musculoskeletal impairments in migraine and TTH patients, such as FHP and cervical range of motion (ROM) [55]. Patients with TTH had more cervical impairments than migraine and healthy controls, while migraine patients, even though reporting NP, had no differences in most assessed cervical outcomes compared to controls [55]. TTH is also usually distinguished from CeH except in the rare cases of bilateral CeH [71]. Supporting evidence for cervicogenic headache includes mechanical precipitation of an attack [45].

Mechanical precipitation of headache is characteristic of CeH, but it is not specific and can happen in migraine [71]. Also, migraine may coexist with CeH [72] and the co-occurrence may increase the number of migraine episodes and analgesic use [73]. Pain location itself may not be enough to distinguish between migraine and CeH as migraine patients can have pain concentrating on occipital and neck regions, and up to 50–72% of patients with CeH report pain in the frontotemporal area [74]. Differences between CeH and migraine include epidemiological–in CeH women are not clearly more affected than men and CeH starts later in life; and clinical findings–unilateral pain is usually side-locked and does not change sides between episodes [71]. One meta-analysis showed that flexion-rotation test/ROM and neck flexion strength may support the diagnosis of cervicogenic headache over migraine [71].

Finally, there is evidence that greater occipital nerve block can improve the treatment response in migraine. However, using nerve blocks alone is not sufficient for the differential diagnosis of these disorders, sine both TTH and CeH may respond to these treatments [75].

There are other headaches that may occasionally present with NP. Occipital neuralgia is a paroxysmal lancinating pain over the greater occipital nerve distribution area that is associated with hypoesthesia or dysesthesias and tenderness on palpation [45]. The pain is expected to disappear following a local anesthetic block. Cluster headache patients may also report cervical pain, however, the diagnosis is usually confirmed by the other clinical findings [76].

### 3.6. Neck Pain among Children with Migraine

Similar to autonomic symptoms, NP appears to be a particular common migraine feature in the pediatric population [38]. As for the adult population, there is data for NP as both part of the migraine and as a trigger. In a prospective study that included 170 pediatric patients with migraine who were referred to a tertiary hospital for neurological assessment, NP or neck stiffness was reported as a premonitory symptom in 41.5% patients [77]. Moreover, almost half of these patients were first referred to another specialty, such as orthopedics or neurosurgery. Interestingly, NP was more frequent among patients with CAS [77]. This is shown by several other studies that suggest that NP is one of the most common premonitory symptoms, as well as fatigue and mood changes.

## 4. Conclusions

Understanding the role of CAS and neck pain in migraine is vital to comprehend the disorder and to appreciate more completely the disability associated with each attack [1]. Clearly, CAS are common in migraine, with aural fullness, lacrimation, facial/forehead sweating, and conjunctival injection being the most frequent ones. Migraineurs experiencing CAS are more likely to have more severe, frequent, and longer attacks, as well as higher rates of photophobia, phonophobia, osmophobia, and allodynia. Recently, a Danish study proposed a set of diagnostic criteria for migraine with CAS and it seems adequate to include them, in the future, in the International Classification of Headache Disorders. CAS occur due to the activation of the trigeminal autonomic reflex and the differential diagnosis, particularly with CH, can be troublesome. Although CH and migraine share some pathophysiological pathways, CAS in migraine tend to be less severe, less consistent, and are usually bilateral.

Evidence shows that NP is frequent in migraine suffers. Musculoskeletal findings are common in migraine patients, however, most findings of impairments in cervical function may be variations of the normal of unclear significance. It is still not completely clear whether NP is part of the migraine prodromal symptoms or if it acts as a trigger of the attack. Likely both are true and may reflect a central sensitization process. The convergence between upper cervical and trigeminal nociception via the trigeminal nucleus caudalis is the likely mechanism for headache resulting from cervical disease as well as NP in primary headaches. Recognizing NP in migraine is important for diagnostic purposes and also for treatment options, as physical therapy interventions may improve clinical symptoms [73]. Our conclusions are summarized in Table 2.

## Figures and Tables

**Table 1:** Prevalence of cranial autonomic symptoms (CAS) in patients with migraine with CAS and cluster headache.

CAS	Migraine with CAS	Cluster Headache
Lacrimation	14–45% [6,7,10,11,12,13,14]	50–91% [11,15,16,17]
Facial sweating/Flushing	6–39% [6,7,11,12,13]	32–56% [11,15,16,17]
Conjunctival injection	2–36% [6,7,10,12,13,14]	52–86% [11,15,16,17]
Eyelid edema	7–43% [7,10,11,12,13,14]	21–74% [11,16,17]
Nasal congestion	9–32% [6,7,10,12,13]	45–75% [11,16,17]
Aural fullness	13–27% [7,12,13]	9% [16]
Rhinorrhea	5–18% [6,7,10,12,13]	41–72% [11,15,16,17]
Ptosis	4–17% [6,7,10,12,13]	15–74% [15,16,17]
Miosis	2–6% [6]	6–29% [15,16,17]

**Table 2:** Conclusions.

Neck pain and CAS are very frequent among migraine patients.
Migraineurs experiencing CAS are more likely to experience more severe, frequent, and longer attacks.
CAS in migraine are less severe, less consistent, and are usually bilateral, when compared to CAS in CH.
In most cases, neck pain is likely part of the migraine phenomenology. However, it can also be a migraine trigger.
The presence of pain in the cervical region in migraine patients should not prompt the need to do cervical spine imaging, especially in patients with typical migrainous features

## Data Availability

Not applicable.
